# Improving the Delivery and Documentation of DVLA Advice for Patients Discharged from Psychiatric Inpatient Units

**DOI:** 10.1192/bjo.2026.11269

**Published:** 2026-06-30

**Authors:** Sunday Adeoye, Odira Anakebe, Raheel Mushtaq

**Affiliations:** Humber Teaching NHS Foundation Trust, Hull, United Kingdom

## Abstract

**Aims:**

Driving is a complex activity that may be significantly impaired by mental illness, acute psychiatric symptoms, cognitive impairment, and the side effects of psychotropic medication. In the UK, the Driver and Vehicle Licensing Agency (DVLA) provides clear guidance regarding fitness to drive for individuals with mental health conditions. Patients are legally responsible for informing the DVLA of any condition that may affect their ability to drive; however, clinicians have a professional duty to ensure that patients are appropriately advised, and that this advice is clearly documented.

Despite this, DVLA advice is often inconsistently delivered and poorly documented in discharge summaries. This creates risks including:

• Patients unknowingly driving when unfit to do so.

• Potential harm to patients and the public.

• Medico-legal risk to clinicians and the organisation.

• Poor continuity of care for GPs and community teams.

The primary aim of this Quality Improvement Project is:

To improve the delivery and documentation of DVLA driving advice for patients discharged from a mental health inpatient unit, thereby reducing medico-legal risk and improving patient safety.

**Methods:**

This project followed the Plan–Do–Study–Act (PDSA) framework.

PDSA Cycle

• Plan: A baseline audit was conducted to retrospectively review discharge summaries for patient discharge on 1–31 August 2025 and showed no patients had DVLA advice given or documented in their discharge summaries.

• Do: A single, targeted intervention was implemented: An additional question was added to the discharge summary template prompting clinicians to consider and document DVLA driving advice. Effecting this change needed high level hospital management approval. The quality improvement idea was presented in different management meetings including Digital delivery group, corporate delivery group and clinical oversight delivery group before this change was approved and implemented.

• Study: Following implementation, a post-intervention survey was conducted to assess the delivery and documentation of DVLA advice. It showed that all the patient discharges in January 2026 so far have DVLA advice given and documented. This is a significant improvement from the pre-intervention survey.

**Act**: We confirm effectiveness and consider sustain ability and wider rollout by implement the change in other clinical areas of the hospital as appropriate.

**Results:**

Overall, the intervention was associated with improved compliance with DVLA guidance and safer discharge practice.

**Conclusion:**

While this project has revolutionised how discharge summary is being written in the Trust by introducing a single and concise way of documenting DVLA advice, a more robust intervention could have been more appropriate, for example creating a different form specifically for DVLA advice on electronic medical records. But this would, however, need management financial approval and more paperwork for clinicians.

